# Association between a body shape index and osteoarthritis: A cross-sectional study using the NHANES data (1999–2020)

**DOI:** 10.1097/MD.0000000000044869

**Published:** 2025-10-03

**Authors:** Zhen Ai, Jing-xuan Cui, Yang Yang, Ding-xuan Liu, Xi Gao

**Affiliations:** a Heilongjiang University of Chinese Medicine, Heilongjiang, China; b First Hospital of Heilongjiang University of Chinese Medicine, Heilongjiang, China.

**Keywords:** a body shape index, males, osteoarthritis, risk factors, sex differences

## Abstract

The relationship between A body shape index (ABSI) and osteoarthritis (OA) remains unclear, particularly regarding sex- and age-related differences. This study aimed to investigate the association between ABSI and OA, as well as its sex-specific and age-related variations. Data from 39,095 U.S. adults in the 1999 to 2020 National Health and Nutrition Examination Survey (NHANES) database were analyzed, with an OA prevalence of 11.50%. ABSI was calculated using the formula: WC (m)/ [BMI (kg/m^2^)^2/3^ × Height (m)^1/2^]. A weighted multivariate-adjusted logistic regression model revealed a significant positive correlation between ABSI and OA: each 0.01 increase in ABSI was associated with a 13% higher risk of OA (OR: 1.13; 95% CI: 1.03, 1.23). Compared with the lowest ABSI quartile, the highest quartile showed a 24% increased risk of OA (OR: 1.24; 95% CI: 1.05, 1.46). Subgroup analyses showed consistent associations between the various interaction responses, but with variations by sex, age, race, and diabetes status. Notably, the association was more pronounced in men and young-to-middle-aged adults (OR for young and middle-aged: 1.17; 95% CI: 1.03, 1.34; OR for men: 1.32; 95% CI: 1.15, 1.53) but not statistically significant in women or older adults. These findings suggest that higher ABSI (especially abdominal obesity) predicts an increased risk of OA in men and young-to-middle-aged populations, warranting targeted interventions. Future studies should use ABSI as a body size assessment tool to explore underlying mechanisms.

## 1. Introduction

Osteoarthritis (OA), the most common chronic degenerative joint disease globally, has shown a marked increase in prevalence due to global population aging and rising obesity rates.^[1]^ According to the World Health Organization (WHO), 10 to 15% of people over 60 years’ experience OA symptoms, with a prevalence of ~10% in men and 18% in women, indicating significant sex differences.^[2]^ Weight-bearing joints such as the knee and hip are most frequently affected, and their pathological progression often leads to joint dysfunction, severely impairing patients’ quality of life and imposing heavy social and medical burdens.^[3]^ The pathogenesis of OA is complex, involving irreversible aging and the combined effects of multiple variable risk factors, including obesity, mechanical loading, metabolic abnormalities, and inflammatory responses.^[3,4]^ Age, a core risk factor, directly accelerates joint degeneration, which rationally explains the rising incidence of age-related OA amid global aging.^[5]^ Additionally, obesity is a key modifiable risk factor in OA development.^[6]^ Weight gain is closely linked to OA progression, as it not only increases mechanical load on articular cartilage but also enhances adipose tissue production of inflammatory factors, such as pro-inflammatory cytokines and adipokines.^[7–9]^ Due to limitations in clinical treatments^[10]^ – including adverse effects of medications, difficulties in patient self-management,^[11]^ and risks of surgical complications^[12,13]^ – accurately identifying the relationship between OA and its associated risk factors, as well as screening for reliable predictors, has become a key focus in addressing this public health issue.

A body shape index (ABSI), proposed by Krakauer et al,^[14]^ is a novel index for assessing abdominal fat, developed using the National Health and Nutrition Examination Survey (NHANES) database to quantify the correlation between body size and health risks.^[15]^ Compared with the traditional body mass index (BMI), ABSI addresses BMI’s limitation of not reflecting body fat distribution, providing a more accurate assessment of abdominal fat, particularly visceral fat.^[16]^ Numerous studies have demonstrated ABSI’s effectiveness in predicting risks of various diseases, including (CVD), cancer, and diabetes.^[17–19]^ Some studies have also confirmed ABSI’s independence in predicting mortality, with no association with other anthropometric variables.^[20]^ Bone health and obesity are closely intertwined: previous studies found a positive association between high-density lipoprotein cholesterol (HDL-C) and osteoporosis risk in obese male populations,^[21]^ while others revealed complex links between obesity and OA – mostly attributed to joint overload from excess weight.^[7]^ However, the causal relationship between abdominal obesity (a key component of obesity) and OA remains unclear. Existing research indicates that abdominal fat, especially central obesity, can both increase local mechanical load and trigger systemic inflammatory responses, promoting articular cartilage degeneration through a dual mechanism.^[8]^ As a more accurate tool for assessing abdominal fat, ABSI may better explain the potential impact of abdominal obesity on OA.

This study aimed to explore the association between abdominal fat and OA using ABSI, with a focus on identifying sex- and age-related differences through subgroup analyses. By leveraging the NHANES database, we investigated the role of ABSI in predicting OA risk, aiming to quantify the impact of abdominal fat on OA with greater precision. Additionally, we sought to reveal sex- and age-specific mechanisms of OA from the perspective of body fat distribution characteristics. The findings are expected to facilitate more accurate interventions targeting OA risk related to body fat distribution and provide a theoretical basis for individualized prevention and treatment strategies tailored to sex and age differences.

## 2. Methods

### 2.1. Study design and population

All participant data were obtained from 10 consecutive cycles (1999–2020) of the NHANES, a large, long-term population health survey database managed by the centers for disease control and prevention and the National Center for Health Statistics (NCHS). This database ensures a representative and diverse sample through sophisticated stratified sampling methods and sampling techniques based on probability clustering. The study design adhered to the requirements of the NCHS Research Ethics Review Board, and all participants provided written informed consent.

**Figure 1.**
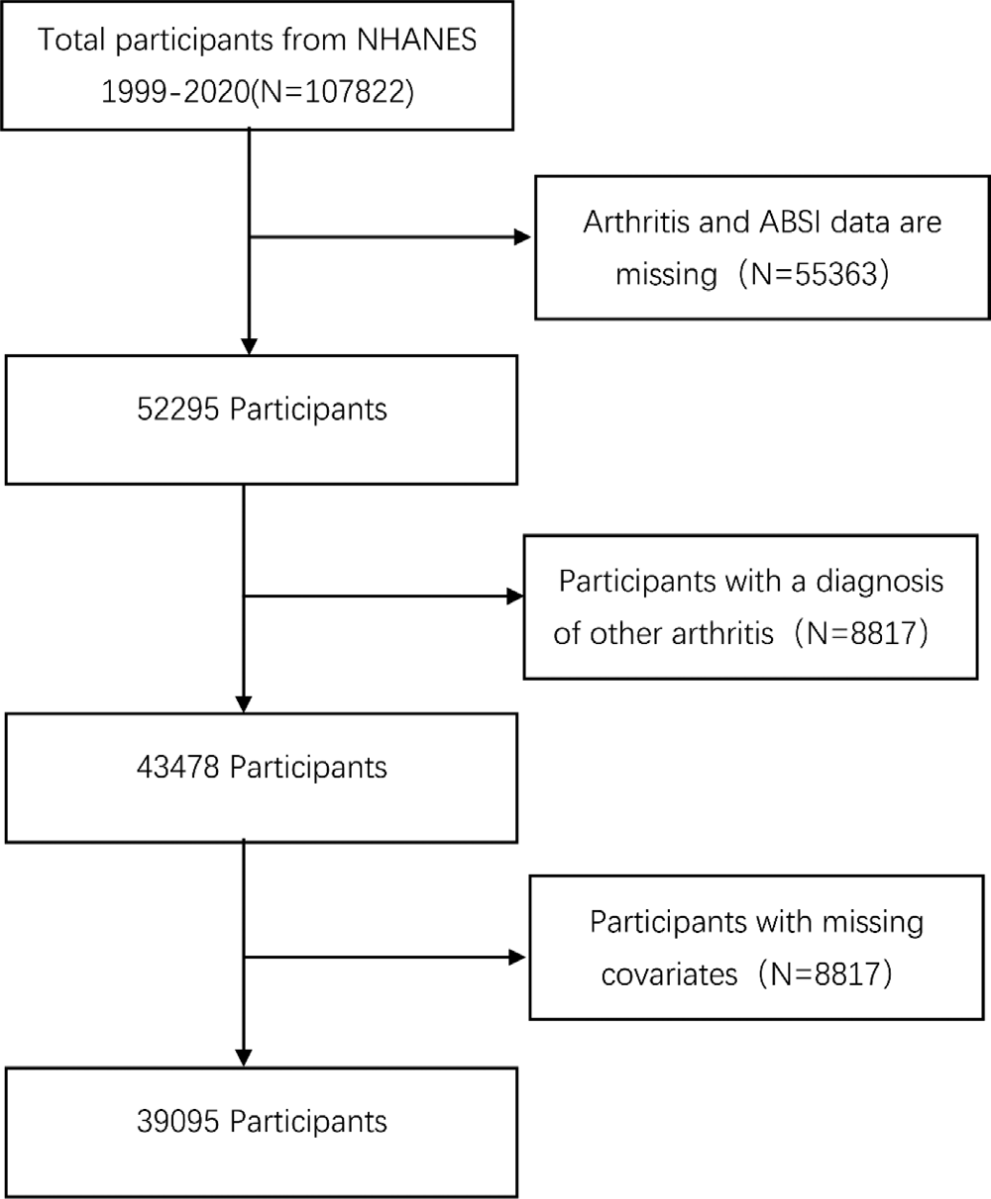
Flow chart of the screening of the NHANES 1999–2020 participants. NHANES = National Health and Nutrition Examination Survey.

Raw data included 107,822 participants across 10 cycles. During data cleaning, participants were excluded if they had missing or incomplete arthritis/ABSI data, a diagnosis of other arthritis types (e.g., rheumatoid arthritis, psoriatic arthritis), or missing/unknown covariate data. After rigorous screening, 39,095 eligible participants were included, with 4494 diagnosed with OA. The detailed screening process is shown in Figure 1.

### 2.2. Definitions of the exposure and outcome variables

All participants underwent waist circumference (WC), height, and weight measurements to calculate ABSI. WC was measured at the highest point of the anterior superior iliac spine during minimal respiration. Height and weight were measured without shoes, in light clothing, using a medical scale. BMI was calculated as weight (kg) divided by height squared (m²). The outcome variable, a history of OA, was determined using 2 self-reported questions from the medical Conditions questionnaire: “Has a doctor or other health professional ever told you that you have arthritis?” and “What type of arthritis is this?” Participants reporting OA as the type of arthritis were selected based on the above 2 questions, while other types of arthritis were excluded from the analysis. The study showed 81% agreement between participants’ self-reported diagnosis of OA and clinical confirmation.^[22]^ The exposure variable was ABSI, and the formula for ABSI was as follows:


$$\begin{array}{l} ABSI=\frac{\left(Waist\;\;Circumference\left(m\right)\right)}{\begin{array}{l} \left(BMI^{\frac{2}{3}}*Height{\left(m\right)}^{\frac{1}{2}}\right)\;\;\; \end{array}} \end{array}$$

### 2.3. Potential covariates

To minimize confounding effects on the association between ABSI and OA, covariates were selected based on previous research and theoretical considerations, including sociodemographic factors and health-related information. Continuous variables included age and poverty income ratio (PIR); categorical variables included sex, race, marital status, education level, smoking status, diabetes, hypertension, and CVD. Information on age, sex (male, female), race (non-Hispanic white, non-Hispanic black, Mexican–American, other Hispanic or other), poverty income ratios, marital status (married/living with partner, widowed/divorced/separated, never married), education level (classified as below high school, high school or above high school), smoking status (Have participants smoked at least 100 cigarettes in their lifetime? Do you smoke now? Categorized as never smoked, ex-smoker, current smoker), diabetes (Yes, No), hypertension (Yes, No), and CVD (Yes, No) were collected through interviews. Detailed measurement methods are available on the NHANES website (http://www.cdc.gov/nchs/nhanes/).

### 2.4. Statistical analysis

Continuous variables in baseline characteristics are presented as means and standard deviations, and categorical variables as frequencies (%). Quartiles of the ABSI group were compared with Student *t* test, Mann–Whitney *U* test and χ^2^ test for categorical variables. Multivariate logistic regression models were used to examine the relationship between ABSI and OA, with 3 models: Model 1 (unadjusted); Model 2 (adjusted for age, sex, and race); and Model 3 (adjusted for age, sex, race, education level, PIR, smoking status, hypertension, diabetes, and CVD). Results are reported as odds ratios (ORs) and 95% confidence intervals (CIs). Generalized additive models and curve fitting were used to visualize the association between ABSI and OA. Stratified analyses and interaction responses were performed to assess interactions between ABSI and covariates in relation to OA. Statistical analyses were conducted using R version 4.4.2 (R Foundation, Vienna, Austria) and EmpowerStats (http://www.empowerstats.com, X&Y Solutions, Inc., Boston). The statistical significance level was set at *P* < .05.

## 3. Results

Table 1 shows the weighted demographic and clinical characteristics of participants stratified by baseline ABSI quartiles. A total of 39,095 participants were included, with 19,223 (49.17%) males and 19,872 (50.83%) females. The mean age ± SD was 46.76 (±17.3) years, and the mean (SD) ABSI was 0.092 (0.006). OA prevalence was 11.50%. ABSI is categorized by quartiles as Q1 (0.0646–0.0876), Q2 (0.0876–0.0921), Q3 (0.0921–0.0965), and Q4 (0.0965–0.1364). All variables were statistically significant across the 4 ABSI subgroups. Compared with the lowest quartile (Q1), participants in Q4 were more likely to be older, better educated, nonsmoking, married or cohabiting, and non-Hispanic white males.

**Table 1:** Basic characteristics of participants by a body shape index quartile.

Variable	ABSI quartiles				*P*-value
	**Q1 (0.0646–0.0876**)	**Q2 (0.0876–0.0921**)	**Q3 (0.0921–0.0965**)	**Q4 (0.0965–0.1364**)	
Age, yr, mean (95% CI)	38.0 (37.5, 38.4)	42.2 (41.7, 42.6)	46.5 (46.1, 46.9)	53.5 (53.1, 54.0)	<.001
Sex, % (95% CI)					
Male	24.3 (23.2, 25.4)	41.9 (40.4, 43.4)	58.4 (57.1, 59.8)	71.6 (70.3, 72.9)	<.001
Female	75.7 (74.6, 76.8)	58.1 (56.6, 59.6)	41.6 (40.2, 42.9)	28.4 (27.1, 29.7)	
Race, % (95% CI)					
Mexican American	8.3 (7.3, 9.4)	10.0 (8.8, 11.3)	9.5 (8.2, 10.8)	5.3 (4.5, 6.3)	<.001
Other Hispanic	7.2 (6.2, 8.3)	6.6 (5.7, 7.5)	5.3 (4.5, 6.2)	3.3 (2.7, 4.1)	
Non-Hispanic White	60.0 (57.7, 62.2)	64.8 (62.5, 67.1)	69.3 (67.0, 71.5)	80.1 (78.3, 81.8)	
Non-Hispanic Black	15.4 (13.9, 16.9)	11.1 (10.0, 12.4)	8.8 (7.9, 9.9)	6.6 (5.8, 7.6)	
Other Race	9.2 (8.4, 10.1)	7.4 (6.7, 8.3)	7.1 (6.3, 8.0)	4.6 (4.0, 5.3)	
Education level, % (95% CI)					
Less than high school	13.4 (12.4, 14.4)	14.8 (13.8, 15.9)	16.0 (14.9, 17.2)	15.1 (14.0, 16.3)	<.001
High school	21.1 (19.8, 22.4)	22.8 (21.5, 24.2)	24.1 (22.9, 25.4)	25.2 (24.0, 26.5)	
More than high school	65.5 (63.8, 67.2)	62.4 (60.5, 64.2)	59.9 (58.2, 61.6)	59.7 (58.0, 61.4)	
Marital status, % (95% CI)					
Married/Living with partner	57.7 (56.2, 59.3)	63.4 (61.8, 64.9)	67.7 (66.3, 69.2)	69.2 (67.7, 70.6)	<.001
Widowed/Divorced/Separated	12.1 (11.3, 13.0)	13.2 (12.4, 14.1)	15.0 (14.0, 16.0)	17.7 (16.6, 18.8)	
Never married	30.2 (28.6, 31.8)	23.5 (22.0, 25.0)	17.3 (16.1, 18.4)	13.2 (12.2, 14.2)	
PIR, mean (95% CI)	3.0 (2.9, 3.0)	3.0 (3.0, 3.1)	3.1 (3.0, 3.2)	3.2 (3.1, 3.2)	<.001
Smoking status, % (95% CI)					
Never	65.3 (63.9, 66.7)	58.7 (57.1, 60.4)	53.2 (51.6, 54.7)	45.0 (43.4, 46.5)	<.001
Former	16.2 (15.2, 17.2)	19.8 (18.5, 21.2)	24.8 (23.6, 26.0)	34.1 (32.9, 35.4)	
Now	18.5 (17.4, 19.8)	21.5 (20.2, 22.8)	22.1 (20.9, 23.3)	20.9 (19.7, 22.2)	
Hypertension, % (95% CI)	14.6 (13.7, 15.5)	21.6 (20.4, 22.9)	29.6 (28.4, 30.9)	41.8 (40.5, 43.2)	<.001
Diabetes, % (95% CI)	2.4 (2.0, 2.9)	4.5 (4.0, 5.1)	7.8 (7.1, 8.5)	14.5 (13.7, 15.4)	<.001
CVD, % (95% CI)	2.7 (2.3, 3.1)	3.9 (3.4, 4.3)	7.0 (6.4, 7.7)	13.2 (12.3, 14.1)	<.001
OA, % (95% CI)	7.6 (7.0, 8.4)	10.3 (9.4, 11.2)	12.9 (12.0, 14.0)	17.9 (16.9, 19.1)	<.001

Mean ± SD for continuous variables: the *P* value was calculated by the weighted linear regression model. (%) for categorical variables: the *P* value was calculated by the weighted χ^2^ test.

ABSI = a body shape index, CVD = cardiovascular disease, OA = osteoarthritis, PIR = poverty-to-income ratio.

### 3.1. Regression analysis

**Figure 2.**
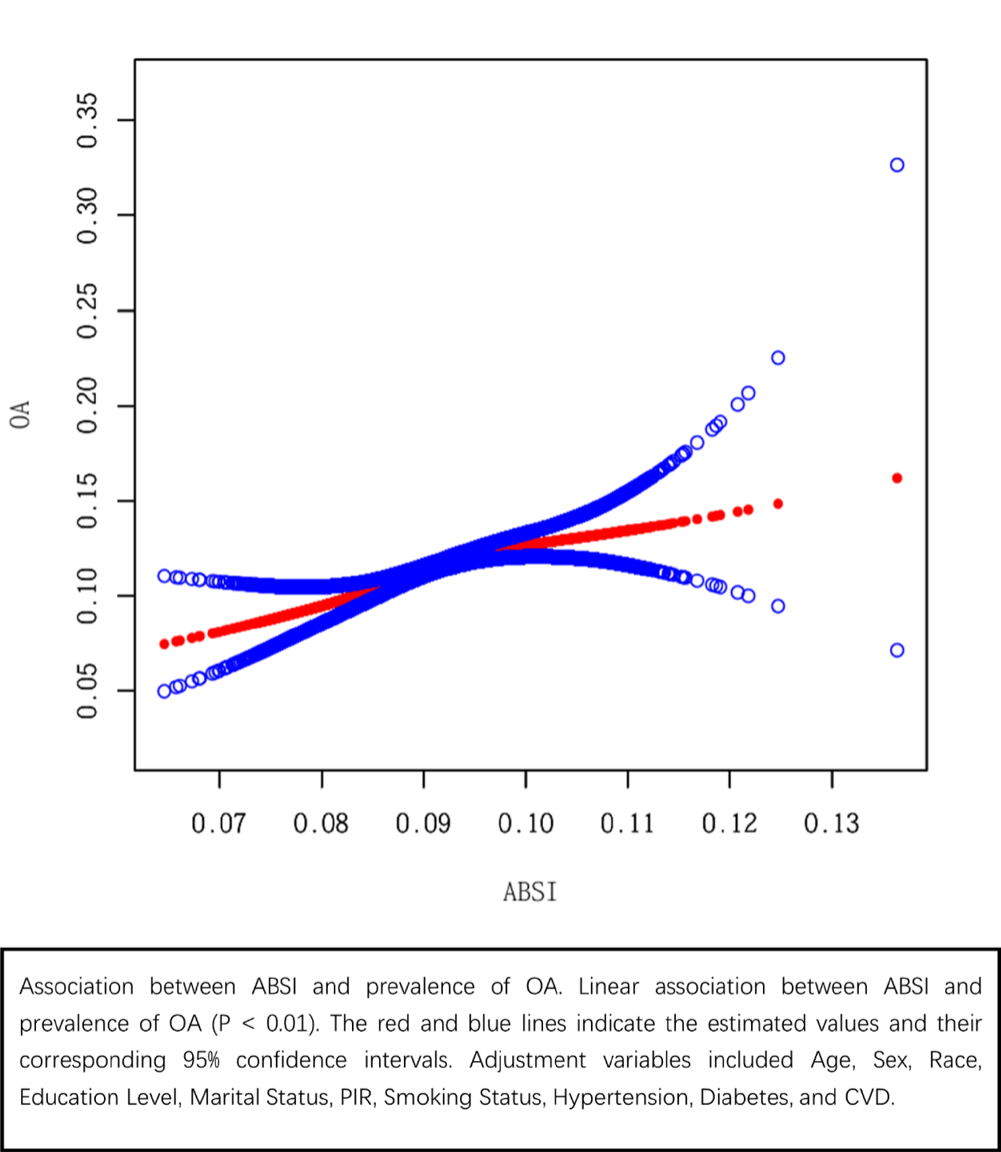
curve fitting: Association between ABSI and prevalence of OA. Linear association between ABSI and prevalence of OA (*P* < .01). The red and blue lines indicate the estimated values and their corresponding 95% confidence intervals. Adjustment variables included age, sex, race, education level, marital status, PIR, smoking status, hypertension, diabetes, and CVD. ABSI = a body shape index, CVD = cardiovascular disease, OA = osteoarthritis, PIR = poverty-to-income ratio.

Table 2 presents multivariate regression analyses of the relationship between ABSI and OA, showing a positive correlation in all participants. In Model 3 (fully adjusted), each 0.01 increase in ABSI was associated with a 13% higher OA risk (*P* < .01). Compared with Q1, Q4 was associated with a 24% higher OA risk (*P* < .05). Trend tests were statistically significant in all models (*P* < .01). Generalized linear models confirmed a significant linear relationship between ABSI and OA (*P* < .01), and multivariate regression results were consistent with curve fitting (Fig. 2).

**Table 2:** Relative odds of OA according to ABSI in different models.

ABSI	OA OR (95% CI)		
	**Model 1 OR (95% CI**)	**Model 2 OR (95% CI**)	**Model 3 OR (95% CI**)
Per 0.01 increment	1.80 (1.69, 1.92)***	1.27 (1.16, 1.38)***	1.13 (1.03, 1.23)**
Quartile			
Q1	Reference	Reference	Reference
Q2	1.38 (1.21, 1.58)***	1.16 (1.01, 1.35)*	1.11 (0.96, 1.30)
Q3	1.79 (1.58, 2.03)***	1.35 (1.18, 1.56)***	1.24 (1.07, 1.43)**
Q4	2.34 (2.34, 2.99)***	1.49 (1.27, 1.74)***	1.24 (1.05, 1.46)*
*P* for trend	<.0001	<.0001	.0078

Model 1 was adjusted for none; Model 2 was adjusted for age, sex, race; Model 3was adjusted for age, sex, race, education level, marital status, PIR, smoking status, hypertension, diabetes, CVD.

ABSI = a body shape index, CVD = cardiovascular disease, OA = osteoarthritis, PIR = poverty-to-income ratio.

* *P* < .05.

** *P* < .01.

*** *P* < .001.

### 3.2. Subgroup analyses

**Figure 3.**
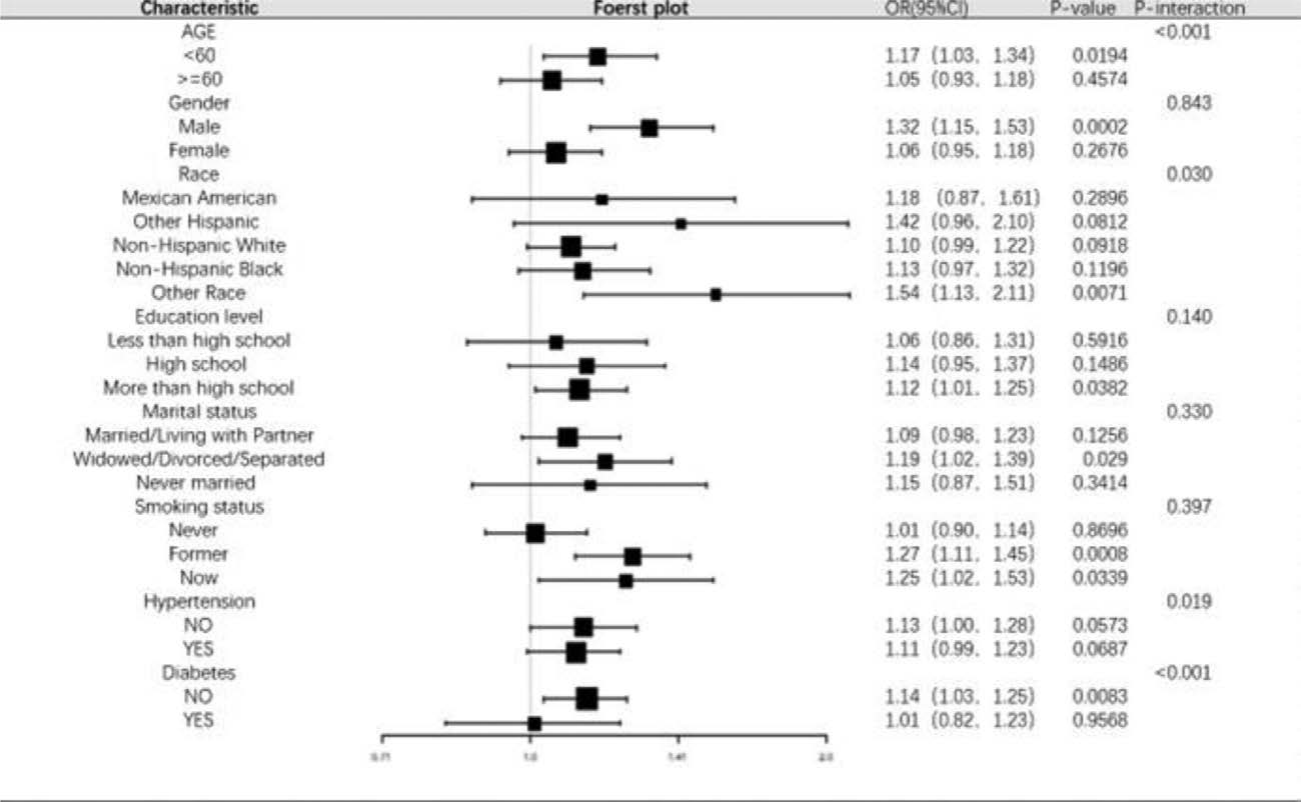
Forest plot.

Subgroup analyses and interaction tests were performed to explore potential associations between ABSI and OA in different populations, including stratification by age (categorized according to the United Nations World Health Organization as: <60 years as young and middle-aged and ≥60 years as older), sex, race, marital status, education level, smoking status, hypertension, and diabetes. Interactions with sex, marital status, education level, and smoking status were non-significant. After full adjustment (Model 3), male participants showed a stronger association (OR: 1.34, 95% CI: 1.15–1.56, *P* < .001), with those in Q4 having a significantly higher OA risk (OR: 1.70, 95% CI: 1.10–2.56, *P* < .05), indicating that higher ABSI increases OA risk in men but not in women (*P* > .05) (Table 3). Age-stratified analysis also showed a significant association: in participants <60 years, higher ABSI was linked to increased OA risk (OR = 1.17, 95% CI: 1.03–1.34, *P* = .019), but this association was non-significant in older adults (*P* = .4574). For hypertension and diabetes, the association between ABSI and OA was significant in participants without hypertension (OR: 1.14, 95% CI: 1.00–1.28, *P* = .0083). Interaction analyses showed that both hypertension (*P* = .019) and diabetes (*P* < .001) modified this association (Fig. 3), suggesting a more complex relationship between ABSI and OA in individuals with hypertension, diabetes, or across different races. These subgroup analyses highlight the value of individualized interventions, with more pronounced effects of ABSI on OA in men and young-to-middle-aged adults, emphasizing the moderating roles of sex and age.

**Table 3:** Relative odds of OA according to ABSI in different models among males and females.

Characteristic	OA OR (95% CI)		
	**Model 1**	**Model 2**	**Model 3**
Male			
ABSI (per 0.01 increment)	3.44 (3.08, 3.83)***	1.48 (1.28, 1.72)***	1.34 (1.15, 1.56)***
Q1 (0.0646–0.0876)	Reference	Reference	Reference
Q2 (0.0876–0.0921)	1.85 (1.15, 2.98)*	1.14 (0.70, 1.85)	1.08 (0.66, 1.77)
Q3 (0.0921–0.0965)	4.14 (2.80, 6.12)***	1.66 (1.10, 2.51)*	1.46 (0.96, 2.23)
Q4 (0.0965–0.1364)	11.16 (9.09, 13.40)***	2.06 (1.34, 3.16)**	1.70 (1.10, 2.65)*
*P* for trend	<.0001	<.0001	<.0001
Female			
ABSI (per 0.01 increment)	2.13 (1.95, 2.33)***	1.21 (1.09, 1.34)***	1.06 (0.95, 1.18)
Q1 (0.0646–0.0876)	Reference	Reference	Reference
Q2 (0.0876–0.0921)	1.71 (1.48, 1.97)***	1.17 (1.00, 1.37)	1.11 (0.95, 1.31)
Q3 (0.0921–0.0965)	2.50 (2.17, 2.88)***	1.17 (1.11, 1.51)***	1.16 (0.99, 1.36)
Q4 (0.0965–0.1364)	3.34 (2.83, 3.93)***	1.33 (1.08, 1.62)**	1.07 (0.87, 1.31)
*P* for trend	< .0001	.0019	.3939

Model 1 was adjusted for none; Model 2 was adjusted for age and race; Model 3was adjusted for age, race, education level, marital status, PIR, smoking status, hypertension, diabetes, CVD.

ABSI = a body shape index, CVD = cardiovascular disease, OA = osteoarthritis, PIR = poverty-to-income ratio.

* *P* < .05.

** *P* < .01.

*** *P* < .001.

## 4. Discussion

Using 10 cycles of NHANES data (1999–2020), we analyzed the association between ABSI and OA, finding a linear positive correlation – particularly significant in men and young-to-middle-aged participants. After adjusting for covariates, higher ABSI was significantly associated with increased OA risk: individuals in Q4 had a 24% higher OA prevalence than those in Q1. Subgroup and interaction tests revealed population-specific differences: the association was stronger in men (non-significant in women) and varied with age, diabetes status, and race, indicating that age, diabetes, and race may modify ABSI’s effect on OA. These findings suggest that ABSI measurement could improve early detection of OA risk, highlighting its potential as a tool for assessing OA risk – especially regarding visceral adiposity – and providing a basis for further exploring the relationship between body size and OA.

In recent years, with rising obesity rates, OA pathogenesis has been increasingly studied.^[23]^ Previous research suggests obesity increases hip or knee OA risk via mechanical factors^[24]^; a Mendelian randomization study found that higher BMI primarily elevates risk in weight-bearing joints, with no causal link to non-weight-bearing joints (e.g., hands).^[25]^ However, BMI, as a measure of overall obesity, has limitations: it is difficult to differentiate between different types of body fat stores and ignores the variability in fat distribution. Belen et al^[26]^ found that OA patients have thicker epicardial fat, which increases with OA severity. Similarly, Toussirot et al^[27]^ found that in patients with OA of the knee, the adipose tissue was significantly thicker and was distributed toward the visceral area. These findings indicate that visceral adipose tissue is not only a marker of obesity but also closely linked to OA onset and progression. A Dutch epidemiology showed^[28]^ that visceral adipose tissue is one of the risk factors for hand arthritis in men (OR: 1.51, 95% CI: 1.13–2.03). Thus, metrics beyond BMI are needed to quantify body fat distribution and assess its impact on bone and joint health. ABSI, which integrates WC, height, and BMI, more comprehensively reflects abdominal fat proportion, offering a more sensitive and specific tool for evaluating the relationship between abdominal obesity and joint function.

Visceral adipose tissue, a secretory organ, produces multiple cytokines that regulate metabolism and inflammation.^[29]^ Inflammation plays a key role in OA: visceral adipose tissue is a major source of pro-inflammatory factors, including tumor necrosis factor-α (TNF-α), interleukin-6 (IL-6), and interleukin-1β (IL-1β).^[30–32]^ These factors, secreted by visceral adipose tissue macrophages, directly impair articular cartilage, synergistically promoting cartilage matrix degradation and bone resorption in OA.^[33]^ Visceral adipose tissue also produces adipokines such as leptin and adiponectin. Several studies report higher leptin and adiponectin levels in OA patients than controls^[34,35]^; another study found leptin partially mediates the obesity-OA link.^[36]^ Griffin et al noted that leptin plays a decisive role in obesity, and that obesity alone is not sufficient to cause OA of the knee.^[37]^ Adiponectin has also been shown to contribute to the degradation and destruction of the cartilage matrix, leading to arthritis and pain.^[38]^ Additionally, visceral fat accumulation can cause metabolic abnormalities, including insulin resistance and elevated blood glucose/lipids.^[29]^ These abnormalities increase free radical production, damaging articular cartilage structure,^[39]^ impairing microvascular function (reducing joint nutrient supply),^[40]^ and stimulating immune responses to produce pro-inflammatory factors, exacerbating joint inflammation.^[41]^

Collectively, these studies indicate that increased abdominal obesity – especially visceral fat accumulation – elevates OA risk. In the present study, we found that in men, participants in the highest ABSI quartile had a higher OA risk (OR = 1.70, 95% CI: 1.10–2.56, *P* < .001), whereas no such trend was observed in women. Our finding of higher ABSI in men aligns with previous studies showing lower abdominal obesity rates (especially visceral fat) in women.^[42]^ This difference may stem from 2 factors: lifestyle: men are more exposed to obesity-promoting behaviors (e.g., smoking, alcohol consumption, high-fat diets), while women often adopt stricter dietary and weight-control practices, reducing obesity prevalence^[43,44]^; biology: male abdominal adipose tissue has high androgen receptor expression, promoting visceral fat accumulation,^[45]^ whereas estrogen – particularly premenopausal – exerts dual protective effects by inhibiting visceral fat synthesis and regulating anti-inflammatory responses.^[46,47]^ Notably, women’s high OA incidence peaks postmenopausal, linked to physiological changes from sudden estrogen decline: estrogen deficiency weakens inhibition of visceral fat accumulation, reduces articular cartilage protection, and exacerbates synovial inflammation.^[48]^ Thus, the onset of OA in women is the result of a combination of age, hormonal fluctuations, local mechanical loads, and systemic metabolic status that cannot be adequately predicted by ABSI alone. In men, occupational weight-bearing, higher sports injury rates, and direct cartilage degradation by visceral fat-derived inflammatory factors form a “abdominal obesity-inflammation-joint degeneration” pathway,^[49]^ strengthening the ABSI-OA association.

Subgroup analyses revealed moderating effects of race, diabetes, and age: the ABSI-OA correlation was strongest in the “other race” subgroup (*P* = .007) with a significant interaction (*P* = .030), suggesting ethnic genetic background may exacerbate joint degeneration by influencing fat distribution, with gene expression enhancing abdominal fat’s role in joint damage.^[50]^ In diabetes-stratified analyses, the association was significant in non-diabetic participants (OR = 1.14, 95% CI: 1.03–1.25, *P* = .008), indicating abdominal obesity increases OA risk in non-diabetic populations. This may be because hyperglycemia in diabetics directly induces chondrocyte glycosylation damage; abnormal glucose regulation may mask abdominal obesity’s effects,^[51]^ reducing ABSI’s predictive value in this group.

Age-related differences were also observed: elevated ABSI was significantly associated with OA risk in adults <60 years but not in those ≥60 years. This aligns with age-related physiological changes: muscle is gradually replaced by adipose tissue after 50 years, and adipose tissue differentiation capacity declines, leading to age-related ABSI increases.^[52,53]^ Increasing age affects the level of hormones in the body.^[54]^ Hormonal changes also play a role: postmenopausal estrogen decline disrupts fat distribution promoting the accumulation of subcutaneous fat^[48]^; male testosterone decreases yearly, weakening visceral fat lipolysis^[55]^; reduced growth hormone secretion inhibits fat mobilization, promoting abdominal fat deposition^[56]^; Concurrently, age-related insulin resistance impairs hepatocyte and adipocyte responses to insulin, increasing glucose-to-fat conversion and visceral fat synthesis.^[57,58]^ These mechanisms make abdominal obesity’s metabolic damage to cartilage more prominent in middle age, while older adults’ multi-factorial joint degeneration (e.g., chondrocyte senescence, reduced matrix synthesis, impaired repair) dilutes ABSI’s independent predictive power.

In summary, multiple mechanisms link abdominal fat accumulation to elevated OA risk, explaining the ABSI-OA association and sex-specific differences. This study is the first to use ABSI as a body image tool to focus on assessing the impact of abdominal obesity on OA. Future studies should explore specific mechanisms, clarifying the abdominal obesity-OA link to enable targeted interventions for fat distribution-related OA risk and reduce obesity-associated OA burden.

This study has several limitations. First, disease diagnoses were identified via self-reported questionnaires in the NHANES database, introducing potential bias; future studies should use clinical diagnoses and medical records for objectivity. Second, the database does not differentiate OA by joint site, precluding detailed site-specific analyses. Third, unmeasured confounders (e.g., diet, exercise) could not be fully excluded. Fourth, NHANES data are U.S.-based, limiting generalizability to other populations. Finally, as a cross-sectional study, it cannot establish causality; longitudinal studies are needed to clarify the ABSI-OA relationship.

## 5. Conclusions

This cross-sectional study of 39,095 U.S. adults found a positive association between ABSI and OA, particularly in men and young-to-middle-aged populations. Sex and age are key moderators of this relationship, indicating that ABSI, as a body size assessment tool, enhances understanding of abdominal fat distribution’s impact on OA. These findings highlight the need to consider individual characteristics when assessing OA risk and designing targeted interventions.

## Author contributions

**Conceptualization:** Zhen Ai.

**Formal analysis:** Zhen Ai, Yang Yang, Ding-xuan Liu.

**Funding acquisition:** Xi Gao.

**Methodology:** Zhen Ai.

**Project administration:** Xi Gao.

**Resources:** Yang Yang.

**Software:** Zhen Ai, Yang Yang.

**Supervision:** Xi Gao.

**Validation:** Jing-xuan Cui, Ding-xuan Liu.

**Visualization:** Yang Yang, Ding-xuan Liu.

**Writing – original draft:** Zhen Ai, Jing-xuan Cui.

**Writing – review & editing:** Zhen Ai.
